# DNA Hypermethylation of the Serotonin Receptor Type-2A Gene Is Associated with a Worse Response to a Weight Loss Intervention in Subjects with Metabolic Syndrome

**DOI:** 10.3390/nu6062387

**Published:** 2014-06-23

**Authors:** Aurora Perez-Cornago, Maria L. Mansego, María Angeles Zulet, José Alfredo Martinez

**Affiliations:** 1 Department of Nutrition, Food Science and Physiology, Center for Nutrition Research, University of Navarra, C/Irunlarrea 1, 31008-Pamplona, Spain; E-Mails: apcornago@alumni.unav.es (A.P.-C.); mlmansego@unav.es (M.L.M); mazulet@unav.es (M.A.Z); 2 CIBER Fisiopatología Obesidad y Nutrición (CIBERObn), Instituto de Salud Carlos III, Santiago de Compostela 15706, Spain

**Keywords:** DNA methylation, *HTR2A* gene, obesity, metabolic syndrome, energy restriction, depressive symptoms, adiposity, epigenetics

## Abstract

Understanding the regulation of gene activities depending on DNA methylation has been the subject of much recent study. However, although polymorphisms of the *HTR2A* gene have been associated with both obesity and psychiatric disorders, the role of *HTR2A* gene methylation in these illnesses remains uncertain. The aim of this study was to evaluate the association of *HTR2A* gene promoter methylation levels in white blood cells (WBC) with obesity traits and depressive symptoms in individuals with metabolic syndrome (MetS) enrolled in a behavioural weight loss programme. Analyses were based on 41 volunteers (mean age 49 ± 1 year) recruited within the RESMENA study. Depressive symptoms (as determined using the Beck Depression Inventory), anthropometric and biochemical measurements were analysed at the beginning and after six months of weight loss treatment. At baseline, DNA from WBC was isolated and cytosine methylation in the *HTR2A* gene promoter was quantified by a microarray approach. In the whole-study sample, a positive association of *HTR2A* gene methylation with waist circumference and insulin levels was detected at baseline. Obesity measures significantly improved after six months of dietary treatment, where a lower mean *HTR2A* gene methylation at baseline was associated with major reductions in body weight, BMI and fat mass after the treatment. Moreover, mean *HTR2A* gene methylation at baseline significantly predicted the decrease in depressive symptoms after the weight loss treatment. In conclusion, this study provides newer evidence that hypermethylation of the *HTR2A* gene in WBC at baseline is significantly associated with a worse response to a weight-loss intervention and with a lower decrease in depressive symptoms after the dietary treatment in subjects with MetS.

## 1. Introduction

Depressive disorders are a significant cause of disability in developed countries and their prevalence is expected to increase in the coming years [1]. This psychiatric disease has been suggested to be often related to the metabolic syndrome (MetS), a state characterized by a combination of central obesity, peripheral insulin resistance, hypertension and serum lipid abnormalities [2,3]. Different theories exist concerning the MetS-depression relationship, but most suggest that it is bidirectional and mediated by several common mechanisms, such as low-grade inflammation, unhealthy dietary habits and genetic factors [4,5].

Indeed, lifestyle and genetic factors affect both depression and MetS [6,7]. Moreover, recent studies have provided evidence that the pathogenesis of these diseases may be also influenced by epigenetic marks [8,9]. In this context, epigenetics is defined as the study of heritable changes in gene expression that, unlike polymorphisms, occur without changes in the DNA sequence and are not permanent [6]. Therefore, epigenetic mechanisms may be capable of explaining some interactions between genetic and environmental factors (e.g., diet, physical activity or drugs), regulating gene expression over the entire lifetime of the organism [10]. Epigenetic changes include multiple processes such as covalent histone modifications, chromatin folding or microRNA abnormalities or DNA methylation of cytosine-phosphate-guanine (CpG) residues, which is probably the issue which has most extensively been studied [6,10]. Whole-genome analysis has often shown an inverse association between DNA methylation and gene expression, especially in the promoter region [11]. Moreover, DNA methylation has been demonstrated to be a predictive tool in the assessment of responses to a nutritional intervention [9,12].

Serotonin (5-HT) is a monoamine neurotransmitter involved in the regulation of important functions such as body temperature, sleep, pain, mood or energy balance [13,14]. Thus, disturbances of the serotonergic pathway have been implicated in both MetS and depressive disorders [13]. There are multiple 5-HT receptors, each with several subtypes and different biological roles [15]. Among them the serotonin 2A receptor, which is encoded by the 5-hydroxytryptamine receptor 2A (*HTR2A*) gene [16], has been involved in the pathogenesis of major psychiatric disorders and obesity [17,18]. In addition to several single nucleotide polymorphisms (SNPs) that have been reported for the *HTR2A* gene [18], DNA methylation factors have also been implicated in the regulation of *HTR2A* gene expression [19]. However, results have been both diverse and inconclusive [16,20,21].

The goal of the present study was to assess the association of *HTR2A* gene promoter methylation levels with obesity measures (e.g., BMI, body weight or fat mass) and depressive symptoms. We tested this hypothesis by determining *HTR2A* gene promoter methylation levels in white blood cells (WBC) of subjects with MetS enrolled in a behavioural weight-loss programme, and then by comparing these methylation levels with both baseline and follow-up obesity traits and depressive symptoms.

## 2. Materials and Methods

### 2.1. Subjects and Study Protocol

The present research is a secondary analysis of the RESMENA (Metabolic Syndrome Reduction in Navarra) project which is a randomized controlled trial, focused on improving MetS features through two dietary strategies (the control and the RESMENA diets) designed for weight loss during a six-month period [9,22,23,24,25]. Both diets were designed following the same energy restriction (−30% of the studied requirements), cholesterol (>300 mg/day) and fibre content (20–25 g/day). The control diet was based on the American Heart Association (AHA) guidelines, including 3–5 meals/day, a macronutrient distribution of 55% total caloric value (TCV) from carbohydrates, 15% proteins and 30% lipids. On the other hand, the RESMENA diet was designed with a higher meal frequency, consisting of 7 meals/day, and a macronutrient distribution of 40% TCV from carbohydrates, 30% proteins and 30% lipids, as described elsewhere [23].

In this analysis within the RESMENA study, 41 Caucasian adults of the two arms of the study were pooled [23,24,25,26]. The study was approved by the Ethics Committee of the University of Navarra (065/2009) and appropriately registered at Clinical Trials.gov; NCT01087086. Each participant provided written informed consent for participation in agreement with the Declaration of Helsinki. This research was performed following the CONSORT 2010 guidelines. This study was conducted in the Metabolic Unit of the University of Navarra in Pamplona, Spain, over a period of 23 months (from January 2010 to November 2011) [23]. Details of the design and methods of this trial have been reported elsewhere [26].

### 2.2. Anthropometry and Blood Pressure

Anthropometric measurements were taken with participants in fasting conditions and wearing only their underwear and using previously validated procedures [26]. Body mass index (BMI) was determined as the body weight divided by the squared height (kg/m^2^). Systolic (SBP) and diastolic (DBP) blood pressures were measured following standardized World Health Organization criteria [27]. Body composition was specifically measured by a dual-energy X-ray absorptiometry (DEXA Lunar Prodigy, GE Medical Systems, Madison, WI, USA). Anthropometric measurements as well as blood pressure determinations were carried out at the beginning and at the end point of the intervention.

### 2.3. Biochemical Analysis

Venous blood samples were taken at baseline and at the end of the study after a 12 h overnight fast period by venipuncture. The EDTA–plasma and serum samples as well as WBC were separated from whole blood by centrifugation at 3500 rpm, 5 °C, 15 min (Model 5804R, Eppendorf, Germany), and were frozen immediately at −80 °C until assay (WBC in buffy-coat with and without Trizol reagent (Invitrogen, Carlsbad, CA, USA) as described elsewhere [9,28,29].

Serum glucose, total cholesterol, triglycerides, and non-esterified fatty acids (NEFAs) were measured in a Pentra autoanalyser C-200 (HORIBA ABX, Madrid, Spain) with commercially available kits. Serum fasting insulin was measured by an enzyme immunoassay kit (Mercodia, Uppsala, Sweden).

### 2.4. Psychological Assessment

Symptoms of depression were assessed at the beginning and at the end of the study using the validated Spanish version of the Beck Depression Inventory (BDI) as published elsewhere [30]. The BDI is a 21-item test that measures the presence and degree of depressive symptoms in respondents. Scores can range from 0 to 63, with a score of 10 or higher indicating moderate depressive symptoms. Question number 19 of the test, relating to weight loss, was discarded from all the analyses given that losing weight is considered a manifestation of depression. However, in our volunteers, it was considered a positive aspect because they were enrolled in a weight loss treatment programme [23].

### 2.5. DNA Isolation and DNA Methylation Study

Genomic DNA from WBC was obtained using the Master Pure kit (Epicenter, Madison, WI, USA), and its quality was assessed with PicoGreen dsDNA Quantitation Reagent (Invitrogen, Carlsbad, CA, USA). A total of 500 ng of DNA were modified using EZ-96 DNA Methylation Kit (Zymo Research Corporation, Orange, CA, UDA) according to the manufacturer’s instructions, thus converting cytosine into uracil.

**Figure 1:**
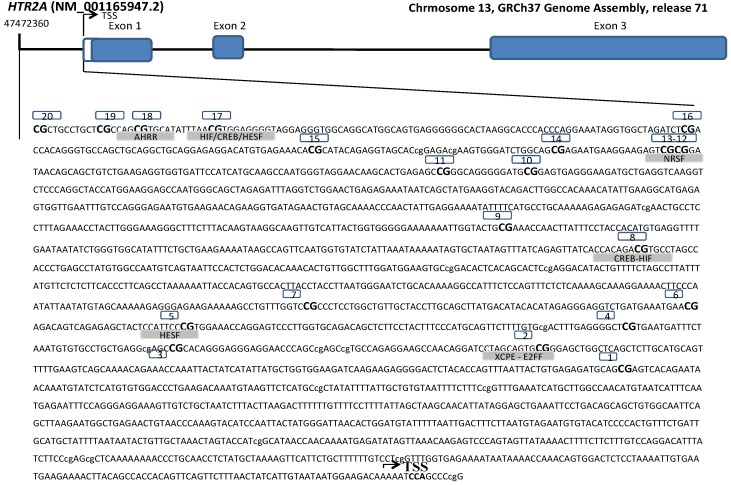
Genomic localization and nucleotide sequence of the 20 CpGs sites covered by the Illumina array for the study of DNA methylation levels of 5-hydroxytryptamine (serotonin) receptor 2A promoter (from −2188 to +10 pb). Transcription Start Site (TSS). Putative consensus sequences for nine transcriptional factors (AHRR: AHR-related factors, HIF: Hypoxia inducible factor, CREB: cAMP-responsive element binding proteins, HESF: Vertebrate homologs of enhancer of split complex (Basic helix-loop-helix domain containing, class B, 2 (secondary DNA binding preference)), NRSF: Neuron-restrictive silencer factor, XCPE: X gene core promoter element 1, E2FF: E2F transcription factor 6, ZF57: KRAB domain zinc finger protein 57, DMTE: *Drosophila* motif ten element), found with MatInspector.

Array-based specific DNA methylation analysis was performed with the Infinium Human Methylation 450K bead chip technology (Illumina, San Diego, CA, USA). Bisulfite-treated genomic DNA was whole-genome amplified, hybridized to HumanMethylation450 BeadChips (Illumina, San Diego, CA, USA) and scanned using the Illumina iScanSQ platform. The intensity of the images was extracted with the GenomeStudio Methylation Software Module (version 1.9.0, Illumina, San Diego, CA, USA). β-Values were computed using the formula β-value = M/(U + M) where M and U are the raw “methylated” and “unmethylated” signals, respectively. β-Values were corrected for type I and type II bias using the peak-based correction. The data were normalized in R using a categorical Subset Quantile Normalization method (SQN) and probes associated to X and Y chromosomes were filtered out using the pipeline developed by Touleimat and Tost [31]. Probes containing SNPs with a minor allele frequency (MAF) >0.001 in Iberian population in Spain were removed from the analysis. The methylation status of 20 CpG sites of the *HTR2A* gene that codes for the HTR2A receptor were selected from the Illumina array and analysed separately. Specific CpG sites located in the transcriptional regulatory region (promoter, 5′-untranslated region and exon 1) were included (Figure 1). Reference names and characteristics of the selected CpG were reported (Table 1).

**Table 1:** Information of the selected CpG sites for *HTR2A* gene.

CpG ID ^1^	Illumina ID	CHR Position ^2^	Reference ^3^
1	cg15894389	13:47470857	c.-688
2	cg02250787	13:47470989	c.-820
3	cg06476131	13:47471052	c.-883
4	cg16188532	13:47471090	c.-921
5	cg09361691	13:47471169	c.-1000
6	cg11514288	13:47471197	c.-1028
7	cg27068143	13:47471264	c.-1095
8	cg10323433	13:47471562	c.-1393
9	cg02027079	13:47471705	c.-1536
10	cg01192538	13:47472050	c.-1881
11	cg01620540	13:47472064	c.-1895
12	cg06020661	13:47472138	c.-1969
13	cg09798090	13:47472140	c.-1971
14	cg24320398	13:47472158	c.-1989
15	cg18200810	13:47472200	c.-2031
16	cg15692052	13:47472250	c.-2081
17	cg24118521	13:47472330	c.-2161
18	cg23881368	13:47472343	c.-2174
19	cg05506829	13:47472349	c.-2180
20	cg07075299	13:47472360	c.-2191

^1^ Studied CpG identifier; ^2^ Genome assembly: GRCh37, Ensemble release 73.37; ^3^ It begins in the first nucleotide of exon 1.

### 2.6. Analysis of Gene Expression by Quantitative Real-Time PCR

Total RNA from WBC was extracted using Trizol reagent (Invitrogen, Carlsbad, CA, USA) according to the manufacturer’s instructions. The integrity of isolated RNA was also evaluated by Experion (BioRad, Hercules, CA, USA), following the manufacturer’s instructions. Briefly, denatured RNA samples (1 μL) were mixed with sample buffer provided, and 6 μL of each mixed sample was loaded in RNA StdSens chips (Bio-Rad) for analysis. In all samples, we also evaluated the RNA quality indicator number (RQI) and this was considered as optimal (ranging from 7.9 to 10). Furthermore, *HTR2A* transcript expression levels were assessed using quantitative real-time RT-PCR (qPCR). cDNA was synthesized from total RNA (1 μg) of the entire cohort individuals using High Capacity cDNA Reverse Transcription Kit with RNase Inhibitor following the manufacturer’s instructions (Life Technologies, Foster city, CA, USA). The transcript levels for *HTR2A* gene and one housekeeper gene were measured using an ABI Prism 7900HT Fast Real-Time PCR system with a 384-well format and TaqMan Gene Expression Assays (Life Technologies, Foster city, CA, USA) (*HTR2A*: Hs01033524_m1 and *GAPDH*: Hs02758991_g1). The ΔΔCT method was used for quantification (ABI) and the fold changes are reported as 2^−ΔΔCT^ [32].

### 2.7. In Silico Sequence Analysis

Human genomic DNA sequences, from 2188 bp upstream to +1 pb of the transcription Start Site (TSS) of the *HTR2A* gene, were downloaded from the NCBI database (GenBank: NG_013011.1, 13:47472360-13: 47469640) [33]. Possible transcription factor-binding sites were predicted on genomic DNA sequences using MatInspector software (Genomatix Software GmbH, Munich, Germany), which is a specifically designed tool for promoter analysis [34].

### 2.8. Statistical Analyses

Results are shown as mean ± SEM. Data normality was determined by the Shapiro-Wilk test. The mean methylation value of the *HTR2A* gene promoter region, including the 20 CpGs, was calculated. Baseline characteristics of participants according to tertiles of mean *HTR2A* gene methylation were compared. Means and SEM for each variable across the *HTR2A* gene methylation tertiles were calculated, with the differences among them assessed using one-way analysis of variance. Pearson correlations were fitted to evaluate the potential correlations of *HTR2A* transcriptional regulatory region methylation with all sites across the promoter region, gene expression and changes in anthropometric measurements and depressive symptoms. Body weight, WC, BMI and fat mass were divided by medians and the differences between groups were analysed using a Student’s t-test. Moreover, multiple testing correction (Benjamini–Hochberg) analyses were performed when appropriate. Statistical analyses were performed using STATA version 12.0 (StataCorp., College Station, TX, USA). *p*-Value < 0.05 was considered as statistically significant.

## 3. Results

The CpG sites were located within the *HTR2A* promoter region, a 2198 bp region positioned from −2188 to +10 (Figure 1). The transcription factor binding sites in the activation regions of the *HTR2A* promoter were screened by using MatInspector. These analyses showed that the promoter region contained a consensus binding motif in the studied CpG sites for seven transcription factors (AHRR, HIF, CREB, HESF, NRSF, XCPE, E2FF) (see Figure 1 and supplementary data S1). Mean DNA methylation levels revealed a high correlation with some of the CpG sites for the HTR2A gene (supplementary data S2). Moreover, DNA methylation levels of CpG sites within the promoter region were associated in some different regions. These distinct areas might imply the presence of specific response elements of gene regulatory machinery in the gene promoter.

The baseline anthropometric, biochemical and psychological characteristics of the whole-study sample (*n* = 41) stratified by tertiles of baseline mean *HTR2A* gene promoter methylation levels are presented (Table 2). In this context, those individuals in the upper category of mean *HTR2A* methylation levels had a higher waist circumference (WC) and insulin levels. Nevertheless, no association between *HTR2A* methylation levels and depressive symptoms was observed at the beginning of the study.

**Table 2:** Anthropometric, biochemical and psychological characteristics of the whole-study sample stratified bytertilesof mean *HTR2A* methylation levels at baseline.

Variables	Mean HTR2A Gene Methylation %			*p* *Trend*
	Low (*n* = 14)	Medium (*n* = 14)	High (*n* = 13)	
Body weight (kg)	97.6 ± 4.6	106.4 ± 4.6	110.7 ± 4.8	0.059
BMI (kg/m^2^)	37.0 ± 1.0	37.0 ± 1.0	37.0 ± 1.0	0.995
Waist circumference (cm)	110.8 ± 3.0	114.0 ± 3.0	120.5 ± 3.1	**0.027**
Total fat mass (kg)	43.8 ± 2.4	45.9 ± 2.4	45.4 ± 2.5	0.682
Truncal fat mass (kg)	26.1 ± 1.8	27.6 ± 1.8	26.2 ± 1.9	0.993
SBP (mmHg)	150.3 ± 4.8	151.2 ± 4.8	148.1 ± 4.9	0.733
DBP (mmHg)	85.4 ± 2.2	87.3 ± 2.2	84.8 ± 2.3	0.784
Glucose (mg/dL)	9.8 ± 1.5	7.4 ± 1.5	11.1 ± 1.6	0.498
Insulin (μU/mL)	11.0 ± 2.3	18.1 ± 2.3	21.7 ± 2.4	**0.004**
TC (mg/dL)	225.1 ± 12.4	211.1 ± 12.9	205.1 ± 12.9	0.280
Triglycerides (mg/dL)	166.8 ± 29.9	224.6 ± 31.1	247.3 ± 31.1	0.079
NEFA (nmol/L)	0.58 ± 0.05	0.50 ± 0.05	0.56 ± 0.05	0.705
BDI score	9.8 ± 1.9	8.3 ± 1.9	10.9 ± 1.9	0.636

Data expressed as mean ± SEM. Bold numbers indicate statistical significance (*P* < 0.05). Abbreviations: BDI, Beck Depression Inventory; BMI, body mass index; DBP, diastolic blood pressure; *HTR2A*, 5-hydroxytryptamine receptor 2A; NEFA, non-esterified fatty acids; SBP, systolic blood pressure; STAI, State Trait Anxiety Inventory; TC, total cholesterol. Low ≤ 0.556; Medium = 0.557–0.585; High ≥ 0.587.

**Figure 2:**
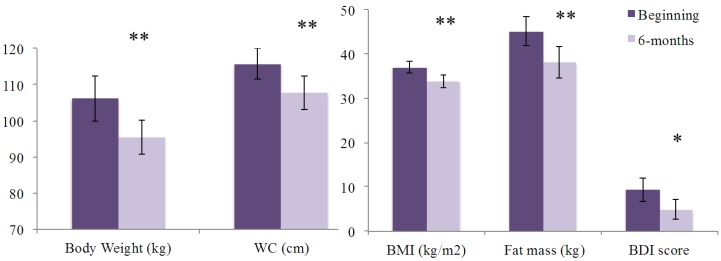
Anthropometric, body composition measurements and in depressive symptoms in the whole-study sample before and after the dietary treatment *n* = 32–34. Data are expressed as mean (CI 95%). Abbreviations: BDI, Beck depression inventory; BMI, body mass index; WC, waist circumference. * *p* < 0.05. ** *p* < 0.001.

**Figure 3:**
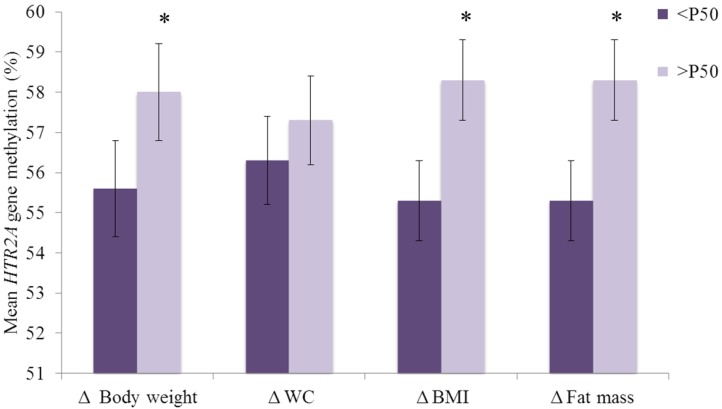
Association of baseline mean *HTR2A* gene methylation (%) with changes (six-month baseline) in anthropometric and body composition measurements divided by their medians in the whole-study sample *n* = 34. Δ = six-month baseline. Data are expressed as mean (CI 95%). Abbreviations: BMI, body mass index; WC, waist circumference. <P50 = high responders; >P50 = low responders to the dietary treatment. * *p* < 0.05.

The 20 selected CpG methylation sites of the *HTR2A* gene showed few associations with baseline body weight, WC, BMI and fat mass (supplementary data S3). After the six-month weight-loss treatment, body weight, WC, BMI, fat mass and depressive symptoms significantly decreased in all subjects (Figure 2). Changes in body weight, WC, BMI and fat mass were divided by medians showing that those participants with a greater response to the dietary treatment had lower *HTR2A* gene promoter methylation levels (Figure 3). Moreover, Pearson correlation analyses between mean *HTR2A* gene methylation levels and changes in anthropometric parameters confirmed these findings, since positive correlations between mean *HTR2A* gene methylation levels and changes in body weight, BMI and fat mass, though not in WC, were found.

Pearson correlation analyses were performed to assess the relationship between baseline *HTR2A* gene methylation levels and changes in body weight, WC, BMI and fat mass after the six-month dietary treatment. The analysis of the 20 selected CpG methylation sites of the *HTR2A* gene showed that CpG 4, CpG 7 and CpG 17 sites were also positively related to changes in body weight, BMI and fat mass (Table 3). However, after applying a multiple comparison correction, the statistical significance was attenuated. In addition, no association between *HTR2A* gene methylation with changes in insulin and triglyceride levels was observed.

**Table 3:** Pearson correlation analyses between changes (six-month baseline) in body weight, BMI and fat mass with the baseline methylation (%) of *HTR2A* gene.

		Δ Body Weight		Δ WC		Δ BMI		Δ Fat Mass (kg)	
Baseline	CpG Sites	*r*	Uncorrected *p*-Value	*r*	Uncorrected *p*-Value	*r*	Uncorrected *p*-Value	*r*	Uncorrected *p*-Value
cg15894389	1	0.110	0.537	−0.117	0.510	0.127	0.474	0.167	0.346
cg02250787	2	−0.063	0.725	−0.083	0.639	−0.063	0.723	0.008	0.963
cg06476131	3	0.284	0.103	0.080	0.652	0.286	0.100	0.381	0.026 *
cg16188532	4	0.379	0.027 *	0.124	0.483	0.383	0.025 *	0.415	0.015 *
cg09361691	5	−0.195	0.268	−0.188	0.287	−0.198	0.261	−0.153	0.386
cg11514288	6	−0.143	0.418	−0.154	0.383	−0.144	0.414	−0.145	0.412
cg27068143	7	0.369	0.032 *	0.116	0.513	0.397	0.020 *	0.382	0.025 *
cg10323433	8	0.292	0.094	0.052	0.769	0.350	**0.042**	0.249	0.155
cg02027079	9	0.011	0.951	0.160	0.365	0.009	0.957	0.042	0.815
cg01192538	10	0.225	0.201	0.193	0.274	0.230	0.190	0.214	0.225
cg01620540	11	−0.070	0.694	−0.251	0.152	−0.014	0.937	−0.073	0.680
cg06020661	12	0.177	0.316	0.206	0.243	0.196	0.267	0.196	0.267
cg09798090	13	−0.042	0.814	−0.054	0.762	−0.037	0.837	−0.059	0.740
cg24320398	14	0.338	0.051	0.266	0.128	0.377	0.028 *	0.343	**0.047**
cg18200810	15	0.310	0.075	0.222	0.206	0.360	0.037 *	0.322	0.063
cg15692052	16	0.091	0.608	−0.025	0.890	0.121	0.494	0.103	0.563
cg24118521	17	0.342	**0.047**	0.280	0.108	0.381	0.026 *	0.414	0.015 *
cg23881368	18	0.297	0.087	0.269	0.124	0.319	0.066	0.291	0.095
cg05506829	19	0.280	0.108	0.252	0.151	0.297	0.088	0.268	0.125
cg07075299	20	0.296	0.089	0.250	0.153	0.307	0.078	0.301	0.084

Data are shown as r and *p* values from Pearson correlations analysis. Δ = 6-month baseline. Abbreviations: BMI, body mass index; *HTR2A*; 5-hydroxytryptamine receptor 2A; WC, waist circumference. * *p*-Value < 0.05 after correcting for Benjamini–Hochberg multiple comparisons.

**Figure 4:**
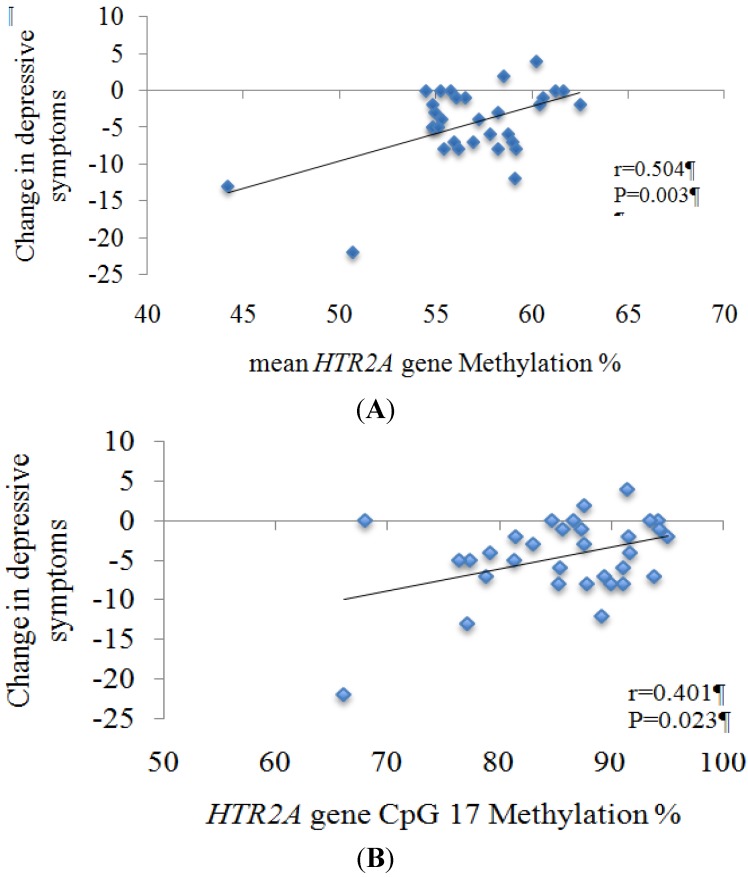
Association of the decrease (six-month baseline) in depressive symptoms with baseline (**A**) mean *HTR2A* methylation; (**B**) methylation levels of *HTR2A* CpG17 site; in the whole-study sample *n* = 32. Change = six-month baseline. Abbreviations: *HTR2A*; 5-hydroxytryptamine receptor 2A.

Interestingly, higher methylation levels of both the HTR2A gene promoter and HTR2A CpG17 site at baseline were related to a less marked decrease in depressive symptoms after the nutritional intervention (Figure 4).

The gene expression analysis showed very low concentrations in WBC and only revealed values for 20 participants. No association was found between gene expression and the *HTR2A* gene promoter methylation levels (*r* = −0.257, *p* = 0.274), or the methylation of *HTR2A* CpG 17 site (*r* = 0.094, *p* = 0.693).

## 4. Discussion

This study showed a positive association between baseline methylation levels of the HTR2A gene in peripheral WBC and both initial and subsequent changes in anthropometric variables, suggesting a regulatory action of this gene on methylation levels in the improvement of obesity measures (e.g., body weight). Moreover, the baseline methylation levels of the *HTR2A* gene promoter region were associated with a decrease in depressive manifestations after the weight loss treatment.

In order to detect putative consensus transcription factor binding sites in the entire *HTR2A* coding region, a computer analysis of this region using MatInspector was carried out [34]. This analysis showed that the *HTR2A* CpG 17 site matches a core-binding motif for CREB, HESF and HIF. A growing literature has related environmental factors to serotonin release acting on diverse serotonin receptors, by the mediation of CREB, which may activate the expression of many genes to produce different proteins involved in neural growth, synapse formation, and long-lasting structural changes, which may be related to depression [20,21]. As for the HESF transcription factor, it has been involved in neuronal excitability in the brain [35]. Moreover, HIF is a key regulator of oxygen homeostasis and has been reported to play a role in the transcriptional regulation of low-grade inflammation, tissue-protective signaling pathways or weight loss [36,37]. Since it has been proposed that low-grade inflammation promotes both obesity and depression [4], it might be hypothesised that methylation of *HTR2A* could interact with obesity and depressive disorders by hindering the binding of HIF, HESF and CREB to the *HTR2A* gene promoter region.

Interestingly, the CpG methylation of a promoter region may be a mechanism implicated in gene inactivation by blocking its transcription [38]. Nonetheless, in this study, we failed to find an association between promoter methylation levels and gene expression, although this might be due to the fact that not all samples could be read. One factor that might explain this unexpected finding is that the promoter region CpG 17 site, an important transcription factor binding site of *HTR2A* gene, was highly methylated and may have caused a very low *HTR2A* gene expression in our sample. Moreover, other studies have found that DNA methylation is involved in the regulation of *HTR2A* expression [19]. On the other hand, a previous study has reported very low concentrations of *HTR2A* gene expression in peripheral blood mononuclear cells, and also an increase of *HTR2A* gene expression after clinical improvement of depressive symptoms [39]. Indeed, this research proposed *HTR2A* gene as a potential biomarker for clinical improvement of depression.

Diet is considered an important environmental factor that affects DNA methylation [6,28]. In this sense, the dietary levels of methyl-donor precursors (vitamins of the B complex or certain amino acids) have been shown to be important in the prevention of psychiatric diseases and obesity [40,41]. In this context, a balance of DNA methylation levels is important for the correct functioning of the central nervous system [40]. Moreover, DNA methylation changes in peripheral WBC have been proposed as a useful biomarker for different diseases [13,38].

On the other hand, one of the most widely studied SNPs of the *HTR2A* gene is rs6311 (−1438G > A), which has been related to many diseases and conditions such as schizophrenia, alcohol dependence, diabetes and obesity [42,43]. For example, it has been suggested that this SNP influences glucose homeostasis [42] and also obesity traits (BMI and abdominal obesity) in obese subjects [44]. In the present study, we found that hypermethylation of the *HTR2A* gene was associated with higher baseline WC and insulin levels. Interestingly, those subjects with higher methylation of *HTR2A* gene and also the *HTR2A* CpG 17 site, showed a less marked decrease in body weight, BMI and fat mass after the six-month dietary treatment. No previous study has analysed DNA methylation levels of *HTR2A* gene in a population with metabolic syndrome enrolled in a weight loss treatment. These results therefore could not be compared. On the other hand, SNPs have not been genotyped in this study. Hence, if the methylation levels are tagging a SNP, this may explain why DNA methylation was not associated with gene expression, as this SNP influences translation efficiency [20,21].

In addition, the *HTR2A* gene has been directly associated with major depressive disorder [45]. Some studies have reported that *HTR2A* expression is decreased in the brain of patients with schizophrenia and in those who have committed suicide [21,46,47]. Moreover, antipsychotic treatment has been linked with lower DNA methylation and increased expression of the *HTR2A* gene [21]. Unfortunately, methylation of cytosines of the *HTR2A* promoter gene in major depressive disorders has not been sufficiently investigated to date [16]. In this sense, it might be suggested that the average methylation of both the *HTR2A* gene and *HTR2A* CpG 17 site predicted the decrease in depressive symptoms.

Under the assumption of an inverse association between DNA methylation and gene expression [38], the density of 5-HT_2A_ receptors might mirror, in part, *HTR2A* methylation. In this sense, lower density of platelet 5-HT_2A_ receptors has been observed in adolescents with eating disorders [48]. Moreover, progressive reductions of brain 5-HT_2A_ receptor density have been proposed as an indicator of schizophrenia [47]. In contrast, results from animal models found that higher 5-HT_2A_ receptor gene expression was linked to obesity [49] and also elevated 5-HT_2A_ receptors in frontal cortex specimens were observed in depressed subjects [50]. Hence, the involvement of 5-HT_2A_ receptors in depressive disorders remains to be elucidated.

Some limitations of the study should be mentioned. Firstly, since the sample size is not very large, the risk of type II errors (failing to detect real differences) was high. Type II errors are frequent when adjustments are used in conjunction with a small effect or a small sample [51]. Therefore, with the aim of avoiding type II errors, no covariates were included in the analysis carried out in this study. Our approach is consistent with previous investigations that reported that when it is important to discover new facts, as is our case, we may be willing to accept more type I errors in order to avoid type II errors [52,53]. Hence, in this study, the probability of type II errors is low because important statistical differences were found despite the small sample size. However, further studies with a large sample size are clearly needed to verify our findings. Secondly, *HTR2A* methylation levels were not measured at the end of the intervention and so it is not possible to confirm whether the dietary treatment had an effect on *HTR2A* gene methylation levels. Thirdly, we failed to find an association between promoter methylation levels and gene expression in WBC. Gene expression varies depending on the tissue or cell type [54], and it is possible that *HTR2A* gene is not widely expressed in WBC. Moreover, other studies have found that DNA methylation is involved in the regulation of *HTR2A* expression [19]. In addition, validation of these results with other reliable standards such as pyrosequencing or bisulfite sequencing might be considered in future studies.

On the other hand, the strengths of this study are: its novelty; the techniques used for DNA methylation quantification, which are known to be the gold standard for epigenetic studies; and its relevance for future dietary intervention trials.

## 5. Conclusions

In summary, this study provides novel evidence that hypermethylation of the *HTR2A* gene in WBC at baseline is significantly associated with a worse response to a weight-loss intervention and a less marked decrease in depressive symptoms in subjects with MetS. These results, if confirmed, would suggest that *HTR2A* gene methylation in WBC could serve as a useful biomarker to predict weight loss and improvement of depressive symptoms after an energy-restricted dietary treatment. However, replication in a larger cohort is warranted in order to confirm these findings and to better understand the possible biological mechanisms explaining these associations.

## Supplementary Files

Supplementary File 1 Supplementary Information (DOCX, 52 KB)
