# Supplementary material for: DNA Hypermethylation of the Serotonin Receptor Type-2A Gene Is Associated with a Worse Response to a Weight Loss Intervention in Subjects with Metabolic Syndrome
Source: Nutrients. 2014 Jun 23;6(6):2387–403. doi: 10.3390/nu6062387 (PMC4073158; doi:10.3390/nu6062387)
Supplement: Supplementary File 1 — Supplementary Information (DOCX, 52 KB) [file nutrients-06-02387-s001.docx]

**Supplementary Information**

**Table S1.** The transcription factors that bind to the region of the *HTR2A* promoter using MatInspector.

| **Matrix Family** ^1^ | **Detailed Family Information** | **Detailed Matrix Information** | **Start Position/End Position** | **Sequence** ^2^ | **CpG ID** ^3^ | | **References** | |
| --- | --- | --- | --- | --- | --- | --- | --- | --- |
| V$AHRR | AHR-arnt heterodimers and AHR-related factors | DRE (dioxin response elements), XRE (xenobiotic response elements) bound by AHR/ARNT heterodimers | 13:47472367/13:47472391 | ctgctcgcca**GCGTg**catatttaac | 18 | | [1–5] | |
| V$HIF | Hypoxia inducible factor, bHLH/PAS protein family | AhR nuclear translocator homodimers | 13:47472383/13:47472399 | atattt**aaCGTG**gaggg | 17 | | [6] | |
| V$CREB | cAMP-responsive element binding proteins | X-box-binding protein 1 | 13:47472382/13:47472402 | catattta**ACGTg**gaggggta | 17 | | [7] | |
| V$HESF | Vertebrate homologues of enhancer of split complex | Basic helix-loop-helix domain containing, class B, 2 (secondary DNA binding preference) | 13:47472385/13:47472399 | attt**aaCGTG**gaggg | 17 | | [8] | |
| V$NRSF | Neuron-restrictive silencer factor | Neural-restrictive-silencer-element | 13:47472571 / 13:47472601 | aag**gaag**a**gtc**g**CGGA**ta**a**c**agc**agctgtct | 13–12 | | [9] | |
| V$HIFF | Hypoxia inducible factor, bHLH/PAS protein family | Hypoxia inducible factor, bHLH/PAS protein family | 13:47473151/13:47473167 | accacaga**CGTG**cctag | 8 | [10–16] | |  |
| V$CREB | cAMP-responsive element binding proteins | X-box-binding protein 1 | 13:47473150/13:47473170 | caccacag**ACGTg**cctagcca | 8 | [7] | |  |
| V$HESF | Vertebrate homologues of enhancer of split complex | Basic helix-loop-helix domain containing, class B, 2 (secondary DNA binding preference) | 13:47473546/13:47473560 | catt**ccCGTG**gaaac | 5 | [8] | |  |
| O$XCPE | Activator-, mediator- and TBP-dependent core promoter element for RNA polymerase II transcription from TATA-less promoters | X gene core promoter element 1 | 13:47473729/13:47473739 | **gtGCGGga**g**c**t | 2 | [17] | |  |
| V$E2FF | E2F-myc activator/cell cycle regulator | E2F transcription factor 6 | 13:47473726/13:47473742 | gcagt**GCGGga**gctggc | 2 | [18–20] | |  |

^1^ The “V$” prefixes to the individual matrices are representative of the Vertebrate MatInspector matrix library, while “O$” prefixes to general core promoter elements. ^2^ Bold: consensus index vector-value > 60 and capital letters: core sequence; ^3^ Studied CpG identifier.

**Table S2.** Pearson correlation analyses between the selected twenty CpG sites for *HTR2A* gene.

| **CpG** | **Mean** | **1** | **2** | **3** | **4** | **5** | **6** | **7** | **8** | **9** | **10** | **11** | **12** | **13** | **14** | **15** | **16** | **17** | **18** | **19** | **20** |
| --- | --- | --- | --- | --- | --- | --- | --- | --- | --- | --- | --- | --- | --- | --- | --- | --- | --- | --- | --- | --- | --- |
| **Mean** | 1.00 |  |  |  |  |  |  |  |  |  |  |  |  |  |  |  |  |  |  |  |  |
| **1** | 0.01 | 1.00 |  |  |  |  |  |  |  |  |  |  |  |  |  |  |  |  |  |  |  |
| **2** | –0.35 | 0.67 | 1.00 |  |  |  |  |  |  |  |  |  |  |  |  |  |  |  |  |  |  |
| **3** | 0.19 | 0.13 | 0.20 | 1.00 |  |  |  |  |  |  |  |  |  |  |  |  |  |  |  |  |  |
| **4** | 0.49 | 004 | -0.05 | 0.62 | 1.00 |  |  |  |  |  |  |  |  |  |  |  |  |  |  |  |  |
| **5** | –0.59 | 0.70 | 0.78 | -0.01 | -0.31 | 1.00 |  |  |  |  |  |  |  |  |  |  |  |  |  |  |  |
| **6** | –0.47 | 0.73 | 0.80 | 0.04 | –0.23 | 0.89 | 1.00 |  |  |  |  |  |  |  |  |  |  |  |  |  |  |
| **7** | 0.73 | –0.28 | –0.68 | 0.21 | 0.41 | –0.60 | –0.70 | 1.00 |  |  |  |  |  |  |  |  |  |  |  |  |  |
| **8** | 0.63 | 0.04 | –0.30 | 0.30 | 0.03 | –0.34 | –0.24 | 0.73 | 1.00 |  |  |  |  |  |  |  |  |  |  |  |  |
| **9** | 0.51 | –0.22 | –0.45 | 0.01 | 0.36 | –0.63 | –0.58 | 0.68 | 0.36 | 1.00 |  |  |  |  |  |  |  |  |  |  |  |
| **10** | 0.65 | –0.62 | –0.78 | 0.08 | 0.40 | –0.97 | –0.89 | 0.82 | 0.43 | 0.70 | 1.00 |  |  |  |  |  |  |  |  |  |  |
| **11** | 0.00 | 0.44 | 0.36 | –0.18 | –0.22 | 0.45 | 0.38 | –0.33 | –0.19 | –0.47 | –0.48 | 1.00 |  |  |  |  |  |  |  |  |  |
| **12** | 0.61 | –0.02 | –0.11 | –0.27 | 0.02 | –0.36 | –0.27 | 0.15 | –0.01 | 0.27 | 0.28 | 0.23 | 1.00 |  |  |  |  |  |  |  |  |
| **13** | –0.16 | 0.64 | 0.67 | –0.27 | –0.37 | 0.71 | 0.69 | –0.61 | –0.33 | –0.44 | –0.76 | 0.64 | 0.34 | 1.00 |  |  |  |  |  |  |  |
| **14** | 0.86 | –0.32 | –0.61 | 0.19 | 0.44 | –0.48 | –0.66 | 0.73 | 0.60 | 0.46 | 0.79 | –0.19 | 0.36 | –0.48 | 1.00 |  |  |  |  |  |  |
| **15** | 0.78 | –0.08 | –0.38 | 0.13 | 0.28 | –0.41 | –0.35 | 0.52 | 0.53 | 0.07 | 0.45 | 0.11 | 0.32 | –0.15 | 0.83 | 1.00 |  |  |  |  |  |
| **16** | 0.43 | 0.37 | 0.22 | –0.24 | –0.06 | 0.17 | 0.19 | –0.10 | –0.03 | –0.03 | –0.20 | 0.60 | 0.70 | 0.71 | 0.12 | 0.32 | 1.00 |  |  |  |  |
| **17** | 0.76 | 0.09 | –0.03 | –0.01 | 0.13 | 0.32 | –0.16 | 0.23 | 0.25 | 0.08 | 0.22 | 0.35 | 0.80 | 0.32 | 0.58 | 0.67 | 0.71 | 1.00 |  |  |  |
| **18** | 0.81 | –0.51 | –0.66 | 0.12 | 0.66 | –0.60 | –0.81 | 0.77 | 0.48 | 0.58 | 0.93 | –0.35 | 0.44 | –0.60 | 0.90 | 0.66 | 0.01 | 0.47 | 1.00 |  |  |
| **19** | 0.76 | –0.56 | –0.75 | 0.11 | 0.45 | –0.95 | –0.84 | 0.79 | 0.46 | 0.62 | 0.96 | –0.41 | 0.40 | –0.67 | 0.88 | 0.60 | –0.07 | 0.39 | 0.98 | 1.00 |  |
| **20** | 0.71 | –0.59 | –0.74 | 0.15 | 0.47 | –0.95 | –0.88 | 0.80 | –0.46 | 0.63 | 0.98 | –0.46 | 0.32 | –0.73 | 0.84 | 0.53 | –0.16 | 0.30 | 0.96 | 0.98 | 1.00 |

Mean = mean *HTR2A* methylation; Flled grey = significantly correlated as identified by Pearson test, *p* < 0.05.

**Table S3.** Pearson correlation analyses between body weight, BMI and fat mass with the methylation (%) of *HTR2A* gene at baseline.

| **Baseline** | **CpG sites** | **Body Weight** | | **WC** | | **BMI** | | **Fat Mass (kg)** | |
| --- | --- | --- | --- | --- | --- | --- | --- | --- | --- |
|  |  | ***r*** | ***P*-Value** | ***r*** | ***P*-Value** | ***r*** | ***P*-Value** | ***r*** | ***P*-Value** |
| cg15894389 | 1 | 0.286 | 0.070 | 0.509 | **0.001** | 0.297 | 0.059 | 0.320 | **0.042** |
| cg02250787 | 2 | 0.207 | 0.195 | 0.393 | **0.011** | 0.327 | **0.037** | 0.294 | 0.062 |
| cg06476131 | 3 | 0.079 | 0.623 | 0.197 | 0.216 | 0.077 | 0.624 | –0.028 | 0.861 |
| cg16188532 | 4 | 0.262 | 0.099 | 0.247 | 0.119 | 0.153 | 0.340 | 0.122 | 0.445 |
| cg09361691 | 5 | 0.180 | 0.260 | 0.350 | **0.025** | 0.355 | **0.023** | 0.327 | **0.037** |
| cg11514288 | 6 | 0.122 | 0.448 | 0.344 | **0.027** | 0.304 | 0.053 | 0.311 | 0.047 |
| cg27068143 | 7 | –0.045 | 0.780 | –0.142 | 0.374 | –0.317 | **0.045** | –0.305 | 0.052 |
| cg10323433 | 8 | 0.002 | 0.987 | –0.018 | 0.910 | –0.339 | **0.030** | –0.268 | 0.090 |
| cg02027079 | 9 | 0.033 | 0.835 | –0.037 | 0.082 | –0.064 | 0.691 | –0.030 | 0.854 |
| cg01192538 | 10 | –0.108 | 0.503 | –0.278 | 0.078 | –0.295 | 0.061 | –0.280 | 0.076 |
| cg01620540 | 11 | 0.139 | 0.384 | 0.241 | 0.130 | 0.057 | 0.725 | 0.250 | 0.120 |
| cg06020661 | 12 | 0.106 | 0.510 | 0.082 | 0.609 | 0.046 | 0.775 | 0.136 | 0.396 |
| cg09798090 | 13 | 0.153 | 0.340 | 0.327 | **0.037** | 0.251 | 0.113 | 0.335 | **0.032** |
| cg24320398 | 14 | 0.017 | 0.913 | –0.082 | 0.612 | –0.288 | 0.067 | –0.255 | 0.108 |
| cg18200810 | 15 | 0.047 | 0.770 | –0.045 | 0.778 | –0.230 | 0.148 | –0.198 | 0.214 |
| cg15692052 | 16 | 0.225 | 0.157 | 0.361 | **0.020** | 0.231 | 0.145 | 0.307 | 0.051 |
| cg24118521 | 17 | 0.137 | 0.393 | 0.137 | 0.394 | –0.026 | 0.871 | 0.012 | 0.941 |
| cg23881368 | 18 | –0.043 | 0.792 | –0.173 | 0.280 | –0.276 | 0.080 | –0.284 | 0.072 |
| cg05506829 | 19 | –0.064 | 0.683 | –0.217 | 0.172 | –0.273 | 0.085 | –0.268 | 0.090 |
| cg07075299 | 20 | –0.078 | 0.626 | –0.239 | 0.133 | –0.289 | 0.067 | –0.301 | 0.056 |

Data are represented as *r* and *p* values from Pearson correlations analysis. Abbreviations: BMI, body mass index; *HTR2A*; 5-hydroxytryptamine receptor 2A; WC, waist circumference.

**References**

1. Wang, F.; Samudio, I.; Safe, S. Transcriptional activation of cathepsin d gene expression by 17beta-estradiol: Mechanism of aryl hydrocarbon receptor-mediated inhibition. *Mol. Cell. Endocrinol.* **2001**, *172*, 91–103.
2. N’Diaye, M.; Le Ferrec, E.; Lagadic-Gossmann, D.; Corre, S.; Gilot, D.; Lecureur, V.; Monteiro, P.; Rauch, C.; Galibert, M.D.; Fardel, O. Aryl hydrocarbon receptor- and calcium-dependent induction of the chemokine ccl1 by the environmental contaminant benzo[a]pyrene. *J. Biol. Chem.* **2006**, *281*, 19906–19915.
3. Patel, R.D.; Kim, D.J.; Peters, J.M.; Perdew, G.H. The aryl hydrocarbon receptor directly regulates expression of the potent mitogen epiregulin. *Toxicol. Sci.* **2006**, *89*, 75–82.
4. Porter, W.; Wang, F.; Duan, R.; Qin, C.; Castro-Rivera, E.; Kim, K.; Safe, S. Transcriptional activation of heat shock protein 27 gene expression by 17beta-estradiol and modulation by antiestrogens and aryl hydrocarbon receptor agonists. *J. Mol. Endocrinol.* **2001**, *26*, 31–42.
5. Augereau, P.; Badia, E.; Fuentes, M.; Rabenoelina, F.; Corniou, M.; Derocq, D.; Balaguer, P.; Cavailles, V. Transcriptional regulation of the human nrip1/rip140 gene by estrogen is modulated by dioxin signalling. *Mol. Pharmacol.* **2006**, *69*, 1338–1346.
6. Swanson, H.I.; Chan, W.K.; Bradfield, C.A. Dna binding specificities and pairing rules of the ah receptor, arnt, and sim proteins. *J. Biol. Chem.* **1995**, *270*, 26292–26302.
7. Clauss, I.M.; Chu, M.; Zhao, J.L.; Glimcher, L.H. The basic domain/leucine zipper protein hxbp-1 preferentially binds to and transactivates cre-like sequences containing an acgt core. *Nucleic Acids Res.* **1996**, *24*, 1855–1864.
8. Badis, G.; Berger, M.F.; Philippakis, A.A.; Talukder, S.; Gehrke, A.R.; Jaeger, S.A.; Chan, E.T.; Metzler, G.; Vedenko, A.; Chen, X.; *et al.* Diversity and complexity in dna recognition by transcription factors. *Science* **2009**, *324*, 1720–1723.
9. Pathak, B.G.; Neumann, J.C.; Croyle, M.L.; Lingrel, J.B. The presence of both negative and positive elements in the 5'-flanking sequence of the rat na,k-atpase alpha 3 subunit gene are required for brain expression in transgenic mice. *Nucleic Acids Res.* **1994**, *22*, 4748–4755.
10. Treins, C.; Giorgetti-Peraldi, S.; Murdaca, J.; Semenza, G.L.; Van Obberghen, E. Insulin stimulates hypoxia-inducible factor 1 through a phosphatidylinositol 3-kinase/target of rapamycin-dependent signaling pathway. *J. Biol. Chem.* **2002**, *277*, 27975–27981.
11. Sanchez-Elsner, T.; Botella, L.M.; Velasco, B.; Corbi, A.; Attisano, L.; Bernabeu, C. Synergistic cooperation between hypoxia and transforming growth factor-beta pathways on human vascular endothelial growth factor gene expression. *J. Biol. Chem.* **2001**, *276*, 38527–38535.
12. Krones, A.; Jungermann, K.; Kietzmann, T. Cross-talk between the signals hypoxia and glucose at the glucose response element of the l-type pyruvate kinase gene. *Endocrinology* **2001**, *142*, 2707–2718.
13. Kakinuma, Y.; Miyauchi, T.; Yuki, K.; Murakoshi, N.; Goto, K.; Yamaguchi, I. Novel molecular mechanism of increased myocardial endothelin-1 expression in the failing heart involving the transcriptional factor hypoxia-inducible factor-1alpha induced for impaired myocardial energy metabolism. *Circulation* **2001**, *103*, 2387–2394.
14. Mazure, N.M.; Chauvet, C.; Bois-Joyeux, B.; Bernard, M.A.; Nacer-Cherif, H.; Danan, J.L. Repression of alpha-fetoprotein gene expression under hypoxic conditions in human hepatoma cells: Characterization of a negative hypoxia response element that mediates opposite effects of hypoxia inducible factor-1 and c-myc. *Cancer Res.* **2002**, *62*, 1158–1165.
15. Narravula, S.; Colgan, S.P. Hypoxia-inducible factor 1-mediated inhibition of peroxisome proliferator-activated receptor alpha expression during hypoxia. *J. Immunol.* **2001**, *166*, 7543–7548.
16. Grosfeld, A.; Andre, J.; Hauguel-De Mouzon, S.; Berra, E.; Pouyssegur, J.; Guerre-Millo, M. Hypoxia-inducible factor 1 transactivates the human leptin gene promoter. *J. Biol. Chem.* **2002**, *277*, 42953–42957.
17. Tokusumi, Y.; Ma, Y.; Song, X.; Jacobson, R.H.; Takada, S. The new core promoter element xcpe1 (x core promoter element 1) directs activator-, mediator-, and tata-binding protein-dependent but tfiid-independent rna polymerase ii transcription from tata-less promoters. *Mol. Cell. Biol.* **2007**, *27*, 1844–1858.
18. Wang, J.; Zhuang, J.; Iyer, S.; Lin, X.; Whitfield, T.W.; Greven, M.C.; Pierce, B.G.; Dong, X.; Kundaje, A.; Cheng, Y.; *et al.* Sequence features and chromatin structure around the genomic regions bound by 119 human transcription factors. *Genome Res.* **2012**, *22*, 1798–1812.
19. Celniker, S.E.; Dillon, L.A.; Gerstein, M.B.; Gunsalus, K.C.; Henikoff, S.; Karpen, G.H.; Kellis, M.; Lai, E.C.; Lieb, J.D.; MacAlpine, D.M.; *et al.* Unlocking the secrets of the genome. *Nature* **2009**, *459*, 927–930.
20. A user’s guide to the encyclopedia of dna elements (encode). *PLoS Biol.* **2011**, *9*, e1001046.

©2014 by the authors; licensee MDPI, Basel, Switzerland. This article is an open access article distributed under the terms and conditions of the Creative Commons Attribution license (http://creativecommons.org/licenses/by/3.0/).
